# Anti-Inflammatory Effects of miR-369-3p via PDE4B in Intestinal Inflammatory Response

**DOI:** 10.3390/ijms25158463

**Published:** 2024-08-02

**Authors:** Viviana Scalavino, Emanuele Piccinno, Nicoletta Labarile, Raffaele Armentano, Gianluigi Giannelli, Grazia Serino

**Affiliations:** National Institute of Gastroenterology S. De Bellis, IRCCS Research Hospital, Via Turi 27, 70013 Castellana Grotte, Italy; viviana.scalavino@irccsdebellis.it (V.S.); emanuele.piccinno@irccsdebellis.it (E.P.); nicoletta.labarile@irccsdebellis.it (N.L.); raffaele.armentano@irccsdebellis.it (R.A.);

**Keywords:** miRNA, IBD, PDE4B, inflammation, miR-369-3p

## Abstract

Cyclic nucleotide phosphodiesterases (PDEs) consist of a family of enzymes expressed in several types of cells, including inflammatory cells, that play a pivotal role in inflammation. Several studies have demonstrated that the inhibition of PDE4 results in a reduced inflammatory response via PKA and CREB signaling. Hence, PDE4 suppression improves the inflammatory feedback typical of several diseases, such as inflammatory bowel disease (IBD). In our previous studies, we have demonstrated that miR-369-3p regulates inflammatory responses, modulating different aspects of the inflammatory process. The aim of this study was to demonstrate an additional anti-inflammatory effect of miR-369-3p targeting PDE4B, one of the widely expressed isoforms in immune cells. We found that miR-369-3p was able to reduce the expression of PDE4B, elevating the intracellular levels of cAMP. This accumulation increased the expression of PKA and pCREB, mitigating the release of pro-inflammatory cytokines and promoting the release of anti-inflammatory cytokines. To prove that PDE4B is a good therapeutic target in IBD, we also demonstrate that the expression of PDE4B was increased in UC patients compared to healthy controls, affecting the immune infiltrate. PDE4B is considered an important player in inflammatory progression; hence, our results show the ability of miR-369-3p to ameliorate inflammation by targeting PDE4B, supporting its future application as a new therapeutic approach in IBD.

## 1. Introduction

Phosphodiesterases consist of several families of enzymes with phosphoric diester hydrolytic cleavage activity. These enzymes include cyclic nucleotide phosphodiesterases (PDEs), phospholipases C and D, autotaxin, sphingomyelin phosphodiesterase, DNases, RNases, and restriction endonucleases [1]. PDEs correspond to the family of enzymes capable of catalyzing the cleavage of cyclic adenosine 3′,5′-monophosphate (cAMP) and cyclic guanosine-3′,5′-monophosphate (cGMP). Through this hydrolytic activity, PDEs regulate the intracellular concentrations of cAMP and cGMP, controlling signaling transduction and, thus, the downstream physiological effects mediated by these secondary messenger molecules [2]. Based on their molecular sequence, kinetics, regulation, and pharmacological characteristics, PDEs are classified into 11 families, namely, PDE1 to PDE11, comprising 21 different gene products [3]. In addition, they may give rise to distinct PDE isoforms as a result of alternative mRNA splicing or transcriptional and translational processing [2,3,4]. Structurally, all PDEs are constituted by a conserved catalytic domain, a regulatory domain at the N-terminus, and a similar C-terminus [3,4]. Functional characteristics of PDEs differ based on their substrate specificities, and according to these, PDE families are subdivided into three main groups: those highly specific for cAMP hydrolysis (PDE4, PDE7, and PDE8), those highly specific for cGMP hydrolysis (PDE5, PDE6, and PDE9), and those with an indifferent affinity for both secondary messengers (PDE1, PDE2, PDE3, PDE10, and PDE11) [4].

The PDE4 family is the largest, consisting of the four genes PDE4A, B, C, and D. Each gene encodes multiple isoforms deriving from alternative splicing or multiple promoters. PDE4s contain a highly conserved catalytic domain, a unique C-terminal region, and an N-terminal region of variable length [3]. This group of enzymes catalyzes cAMP, a secondary messenger that plays a key role in the inflammatory cascade regulating immune functions [5]. PDE4 has been shown to be an effective therapeutic target for distinct inflammatory conditions, including asthma, chronic obstructive pulmonary disease (COPD), psoriasis, atopic dermatitis (AD), inflammatory bowel disease (IBD), rheumatic arthritis (RA), and neuroinflammation [5,6,7].

Inflammatory bowel disease (IBD), including Crohn’s disease (CD) and ulcerative colitis (UC), is an idiopathic disorder characterized by a complex chronic inflammatory state of the gastrointestinal (GI) tract. The etiology, severity, and progression of this disorder are influenced by multiple factors, including genetic predisposition, altered gastrointestinal microbiota, impaired mucosal barrier, deregulated immunological response, and environmental and lifestyle factors [8]. In IBD patients, chronic inflammation is associated with an overproduction of pro-inflammatory cytokines by immune cells [9]. In fact, the relevance of cytokines in the pathophysiology of IBD has led to the development of antibody therapies against cytokines and their signaling cascade [9,10]. An increased activity of PDE4 leads to overproduction of pro-inflammatory cytokines and chemokines in both inflammatory and epithelial cells. Several studies have demonstrated that the inhibition of PDE4 gives rise to an accumulated intracellular level of cAMP, reduces the production of tumor necrosis factor-α (TNF-α), and promotes the expression of interleukin (IL-)10 via protein kinase A (PKA) signaling [5,11,12].

MicroRNAs (miRNAs) are small molecules of non-coding RNA (around 22–24 nucleotides long) able to interact with the 3′ untranslated region (3′ UTR) of target mRNAs, inducing mRNA degradation and translational repression [13]. miRNAs are implicated in numerous physiologic processes, but they also play a crucial role in various diseases, including IBD [14,15]. miRNAs have a key role as regulators of cellular and molecular mechanisms of innate and adaptive immune responses, and their altered expression in immune cells may contribute to the pathogenesis of inflammatory processes [16,17]. In our previous studies, we showed that miR-369-3p was able to regulate inflammatory responses, modulating different aspects of the inflammatory process. Indeed, we demonstrated that miR-369-3p was involved in the modulation of activation of NF-kB signaling pathways through the regulation of C/EBP-β and NOS2 and the subsequent release of pro-inflammatory cytokines [18,19]. Additionally, this miRNA was able to modulate the formation of the immunoproteasome complex through the regulation of the PSMB9/LMP2 subunit and also the assembly and activation of the inflammasome, affecting the deubiquitination of NLRP3 by reducing the BRCC3 expression [19,20].

In this work, we aimed to demonstrate an additional anti-inflammatory property of miR-369-3p acting through the regulation of the PDE4B expression. We demonstrated that in inflammatory conditions, miR-369-3p was able to reduce the expression of PDE4B, triggering an intracellular increase in cAMP. As a result, we found a reduction in pro-inflammatory mediator release and an increase in anti-inflammatory mediator production. These results highlight the anti-inflammatory action of miR-369-3p and hence its promising use in inflammatory diseases. 

## 2. Results

### 2.1. In Silico Analysis of miR-369-3p Gene Targets

**Figure 1:**
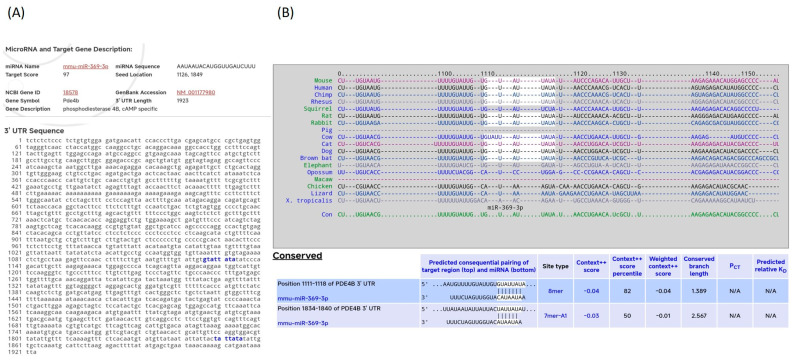
In silico analysis of predicted target interaction between miR-369-3p and Pde4b mRNA. (**A**) miR-369-3p regulates *Pde4b* mRNA expression, aligning with the 3′UTR region through two binding sites. (**B**) The complementary regions highlighted in white of miR-369-3p and *Pde4b* 3′UTR are well conserved among the species.

Firstly, we conducted a bioinformatics analysis to identify new genes targeted by miR-369-3p. Through this in silico analysis, we found that miR-369-3p could regulate *Pde4b* expression by binding two sites of the 3′untranslated region (UTR) (Figure 1A). These two binding sites were well conserved among the species (Figure 1B). 

### 2.2. PDE4B Modulation by miR-369-3p 

**Figure 2:**
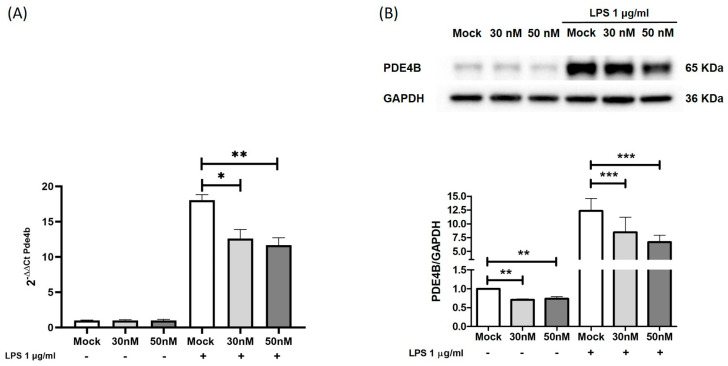
Modulation of PDE4B expression by miR-369-3p. (**A**) The mRNA expression levels of *Pde4b* evaluated by qRT-PCR in Raw264.7 cells transfected with 30 and 50 nM of miR-369-3p mimic before and after LPS stimulation. (**B**) Western blot analysis of PDE4B protein expression after miR-369-3p mimic transfection in unstimulated cells and following LPS stimulation. GAPDH was used as a housekeeping protein to normalize the data. Data are representative of four independent experiments. The histograms correspond to mean ± SEM (* *p* < 0.01; ** *p* < 0.001; *** *p* < 0.0001).

In order to validate this bioinformatic prediction, we carried out in vitro studies using the Raw264.7 cell line. Cells were transiently transfected with mimic molecules of miR-369-3p at two different concentrations of 30 and 50 nM, followed by LPS stimulation. Compared to unstimulated conditions, in LPS-treated cells, the Pde4b gene expression was increased. However, as demonstrated by real-time PCR, the intracellular increase in miR-369-3p significantly decreased the *Pde4b* expression at both mimic concentrations compared to the mock control (*p* < 0.01; Figure 2A). 

Moreover, we also evaluated whether miR-369-3p was able to modulate the PDE4B protein expression. Western blot analysis showed that raising the intracellular miR-369-3p level resulted in a reduction in PDE4B protein levels compared to the mock control, both under basal conditions and after LPS stimulation (*p* < 0.01; Figure 2B). 

**Figure 3:**
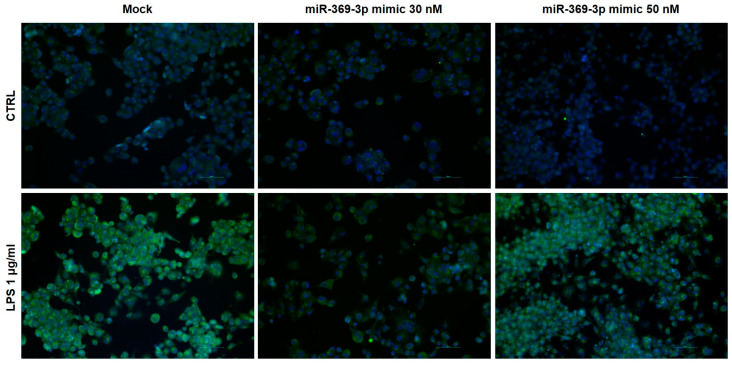
Immunofluorescence staining of PDE4B in Raw264.7 cells after miR-369-3p mimic transfection. In accordance with Western blot results, representative images showed that miR-369-3p modulated the expression of PDE4B compared to the mock control in both unstimulated and LPS-stimulated conditions. For each sample, three images were captured in different positions. Original magnification, 20×. Scale bar, 50 μm.

In addition, to further demonstrate the regulation of PDE4B expression by miR-369-3p, we evaluated the protein expression by immunofluorescence analysis. As shown by immunofluorescence staining, the immunoreactivity was detected in the cytoplasm. In accordance with Western blot results, the images showed that the expression of PDE4B increased after LPS stimulation. Moreover, transient transfection with miR-369-3p mimic decreased the protein expression at both the 30 and 50 nM concentrations compared to the mock control (Figure 3).

### 2.3. miR-369-3p Regulates cAMP Expression

**Figure 4:**
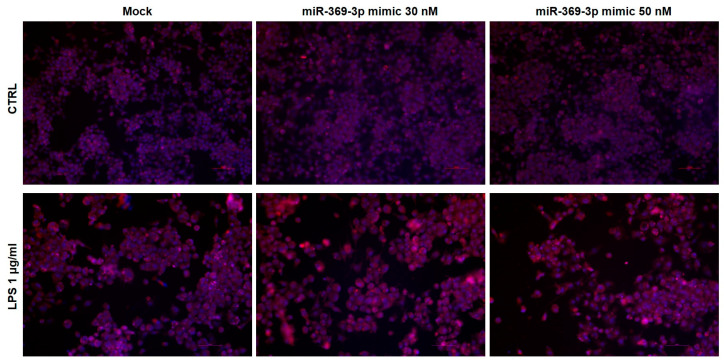
Immunofluorescence staining of cAMP in Raw264.7 cells after miR-369-3p mimic transfection. Representative images of cAMP expression in Raw264.7 cell line transiently transfected with miR-369-3p and stimulated with LPS. For each sample, three images were captured in different positions. Original magnification, 20×. Scale bar, 50 μm.

Considering that PDE4B regulates intracellular concentration of cAMP [21], we investigated the expression of cAMP after miR-369-3p transfection by immunofluorescence staining. In control conditions, without LPS, no differences were observed after miR-369-3p transfection (Figure 4). Following stimulation with LPS, the expression of cAMP increased at both the 30 and 50 nM concentrations compared to the mock control (Figure 4). 

### 2.4. miR-369-3p Affects the Downstream PDE4B Signaling Pathways

**Figure 5:**
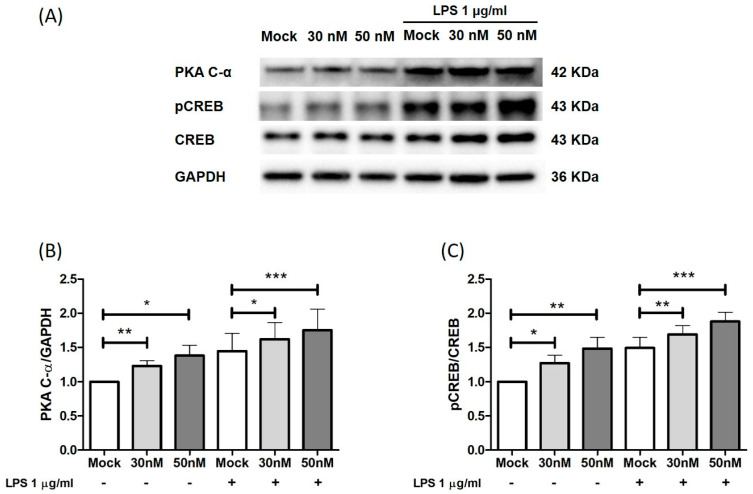
Transient transfection with miR-369-3p affects the downstream PDE4B signaling pathway. (**A**) Representative blots of PKA C-α, pCREB, and CREB protein expressions after miR-369-3p mimic transfection in Raw264.7 cells without and with LPS stimulation. Western blot quantitative analysis of PKA C-α (**B**) and pCREB/CREB (**C**) expressions after miR-369-3p mimic transfection at 30 and 50 nM in unstimulated and LPS-stimulated cells. GAPDH was used as a housekeeping protein to normalize the data. Data are representative of four independent experiments. The histograms correspond to the mean ± SEM (* *p* < 0.05; ** *p* < 0.01; *** *p* < 0.001).

Increased intracellular levels of cAMP activate PKA, which consequently phosphorylates the transcription factor of cAMP response element-binding protein (CREB), leading to the transcription and release of anti-inflammatory cytokines and inhibiting the secretion of pro-inflammatory cytokines [6,21]. Through Western blot analysis, we therefore assessed the effect of miR-369-3p induction on PKA and CREB protein expression (Figure 5A). As displayed in Figure 5, the modulation of PDE4B by miR-369-3p led to an increased expression of PKA in basal conditions (*p* < 0.05; Figure 5B) as well as in LPS conditions (*p* < 0.05; Figure 5B). Similarly, after miRNA transfection, the protein expression levels of pCREB were increased both under unstimulated conditions (*p* < 0.05; *p* < 0.01; Figure 5C) and LPS-stimulated conditions (*p* < 0.01; Figure 5C). 

### 2.5. Effects of miR-369-3p on Anti-Inflammatory and Pro-Inflammatory Cytokine Release

An increased activity of PDE4B leads to the overproduction of proinflammatory cytokines, which contribute to further activation and infiltration of immune cells. The inhibition of PDE4B leads to the intracellular accumulation of cAMP, reduces the release of pro-inflammatory cytokines, and promotes the synthesis of anti-inflammatory cytokines [5,22,23].

**Figure 6:**
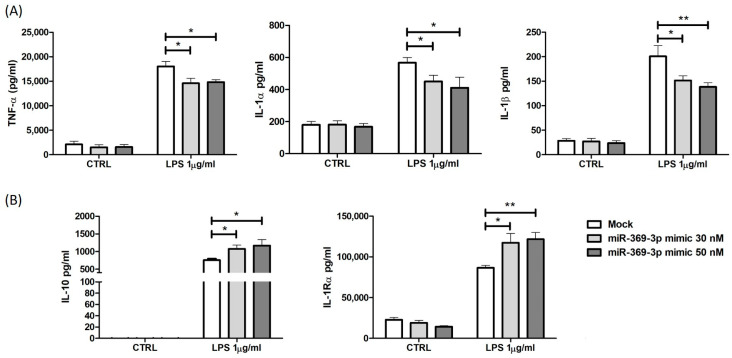
miR-369-3p modulates the release of pro-inflammatory and anti-inflammatory cytokines. The intracellular increase in miR-369-3p in Raw264.7 after transient transfection results in a significant decrease in TNF-α, IL-1α, and IL-1β (**A**) and a significant increase in IL-10 and IL-1Rα (**B**) production in response to LPS stimulation. Data are representative of four independent experiments (* *p* < 0.05; ** *p* < 0.01).

We investigated whether the modulation of PDE4B expression by miR-369-3p was also associated with a control action on pro- and anti-inflammatory cytokine production. After miR-369-3p mimic transfection, cells were stimulated with LPS for 24 h, and the levels of cytokine production were analyzed. miRNA induction significantly decreased the release of TNFα, IL-1α, and IL-1β in response to LPS (*p* < 0.05; *p* < 0.01; Figure 6A). Furthermore, after LPS stimulation, the cellular increase in miR-369-3p increased the production of anti-inflammatory cytokines such as IL-10 and IL-1Rα compared to the mock condition (*p* < 0.05; *p* < 0.01; Figure 6B).

### 2.6. PDE4B Expression in IBD Patients

**Figure 7:**
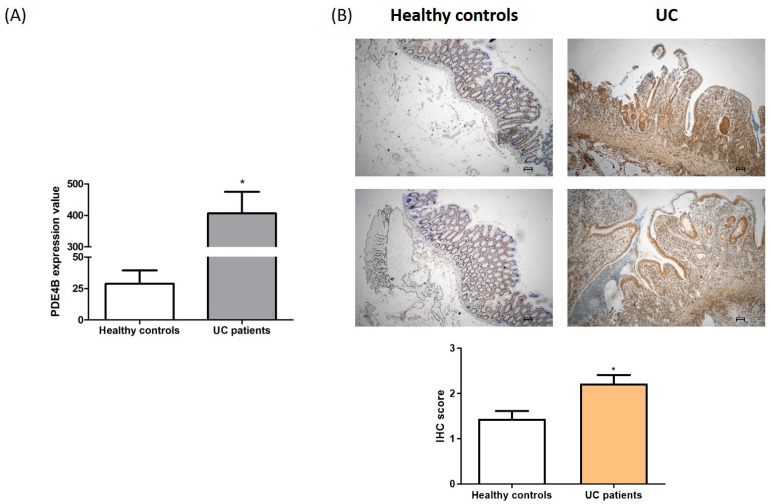
PDE4B expression in UC patients. (**A**) Analysis of gene expression profiles obtained from mucosal biopsies in actively inflamed mucosa from UC patients and normal mucosa from healthy controls downloaded from the GEO database (GSE16879). (**B**) Immunohistochemical analysis of PDE4B protein expression in formalin-fixed, paraffin-embedded tissues obtained from healthy controls and patients with active UC. Original magnification, 4×. Scale bar, 100 μm (* *p* < 0.05).

To further support the concept that PDE4B could be a therapeutic target in IBD, we analyzed mRNA expression from a Gene Expression Omnibus database (GSE16879). The gene expression profiles were obtained from mucosal biopsies in actively inflamed mucosa from ulcerative colitis patients (UC; *n* = 24) and normal mucosa from healthy controls (HC; *n* = 12) [24]. Evaluating the gene expression profile of PDE4B, we found that its expression was significantly increased in the UC group compared to healthy controls (*p* < 0.05; Figure 7A). 

To validate this result, we analyzed PDE4B protein expression in a cohort of tissue specimens from UC patients and healthy controls collected in our institute. PDE4B expression was upregulated in the tissues of UC patients compared to healthy controls, with a diffuse expression in the immune infiltrate (Figure 7B). An inflammation score was used to define the grade of immunostaining. We found that in UC patients, PDE4B expression was significantly higher than in healthy controls (*p* < 0.01; Figure 7B).

## 3. Discussion

IBD is an idiopathic disorder caused by chronic and excessive inflammation of the gastrointestinal tract as a consequence of a dysregulated immune inflammatory state [9,10]. Over the years, biological therapy, such as anti-TNF-α, has emerged as the best instrument for the treatment of IBD [25]. However, a significant number of patients do not properly respond to biological therapies, so it is increasingly necessary to seek new pharmacological targets.

miRNAs play a pivotal role in regulating a multitude of cellular processes, and their differential expression has been related to various human diseases, including inflammatory disorders [26,27,28]. Several reports have found that miRNA deregulation is related to pathways commonly involved in IBD pathogenesis [26,29]. In fact, miRNAs contribute to the maintenance of barrier function in the intestinal epithelium through the regulation of protein expression involved in epithelial barrier integrity [30]. Furthermore, miRNAs may influence the action of immune cells in response to inflammatory signaling related to IBD [16,18,19,20,26].

Phosphodiesterase 4 (PDE4) consists of a group of enzymes that regulate the concentration of cAMP in several types of cells, including inflammatory cells, and plays a role in the inflammatory cascade. Previous studies have demonstrated that high expression levels of PDE4 are involved in inflammatory disorders and in the development of several types of cancer [6,31]. PDE4 also offers a good target for an effective therapeutic strategy for IBD treatment. In fact, in inflammatory cells, PDE4 inhibitors are able to suppress the release of pro-inflammatory mediators and stimulate the release of anti-inflammatory mediators [22,23]. Moreover, these inhibitors ameliorate gut inflammation in animal models of IBD, including mouse models, showing beneficial effects [5].

In the present study, we demonstrated an additional anti-inflammatory property of miR-369-3p in the regulation of the immune inflammatory response. Our in silico analysis showed that PDE4B is a putative target of miR-369-3p. Because PDE4B is one of the widely expressed isoforms in immune cells and is involved in regulating the inflammatory immune response [5,32], we conducted functional studies using the macrophage cell line Raw264.7. We found that the increase in miR-369-3p reduced PDE4B mRNA and protein expression. PDE4B was identified as cAMP-hydrolyzing in human inflammatory cells, thus playing a key role in the regulation of innate immune functions [33]. Under inflammatory conditions, the activation of cAMP signaling has negative effects on immune and inflammatory responses; thus, acting on the cAMP levels is a promising approach for the treatment of chronic inflammatory conditions such as IBD [33,34]. The inhibition of PDE4B can elevate intracellular levels of cAMP, with consequent broad anti-inflammatory effects [12,22,33]. Our results showed that PDE4B modulation by miR-369-3p increased the intracellular levels of cAMP. The accumulation of intracellular cAMP in turn activated PKA and increased the phosphorylation of CREB, leading to a reduction in inflammatory cytokines and an increase in anti-inflammatory cytokines [6,22,23]. This outcome was also reported in our analysis. In fact, we showed that the intracellular increase in cAMP due to modulation by miR-369-3p resulted in an increase in PKA and pCREB. Furthermore, the inhibition of PDE4 was associated with broad anti-inflammatory activity by modulating cytokine release [22,23,34]. Effectively, the action of miR-369-3p suppressed the release of pro-inflammatory cytokines (TNF-α, IL-1β, and IL-1α) and promoted the release of anti-inflammatory mediators (IL-10 and IL-1Rα). The pathological role of PDE4B was also shown in inflammatory colonic tissues from UC patients. We demonstrated that in UC patients, the expression of PDE4B was increased compared to healthy controls at both mRNA and protein levels. Furthermore, we revealed that the high expression of PDE4B in UC patients was mainly identified in the immune infiltrate. An elevated expression of PDE4B in the immune infiltrate is associated with the promotion of inflammatory progression in IBD [35].

PDE4 targeting is a promising clinical approach as it targets a central pathogenic process that impedes complex antigen receptor-specific immunoregulatory mechanisms. Indeed, the inhibition of PDE4 enzymes has been widely studied, and several agents have been evaluated in clinical studies. Roflumilast, the first approved drug inhibitor, is used for the treatment of airway disease [36]. Apremilast is applied for the treatment of dermatological diseases characterized by inflammation [11]. In a pilot study, dipyridamole restored immune homeostasis and alleviated intestinal inflammation in pediatric colitis onset [37]. In a phase II study, apremilast was administered for the treatment of UC patients, and treatment outcomes were promising, even though the end point of the study was not reached and no clinical data are reported about this study [38]. Poor tolerability or effectiveness may be attributable to the early discontinuation of drug administration to UC patients [39]. Other PDE4 inhibitors (rolipram, mesopram, and tetomilast) showed promising therapeutic effects in experimental colitis, but human studies did not reveal the same results [12,40,41].

Due to the serious gastrointestinal adverse effects, investigations on the effect of PDE4 inhibitors in IBD have been slower than those on inflammatory airways and skin diseases. Hence, molecules that are highly specific for PDE4B could improve their therapeutic function in inflammation by minimizing the adverse effects related to the drugs. In this context, miRNA-based therapy could be an optimal strategy to inhibit PDE4 enzymes, minimizing adverse reactions. Here, we demonstrated that in inflammatory conditions, miR-369-3p reduced the expression of PDE4B, triggering an intracellular increase in cAMP. As a result, a reduction in pro-inflammatory mediator release and an increase in anti-inflammatory mediator production were observed. These results highlight the anti-inflammatory action of miR-369-3p and hence its promising use in inflammatory diseases. However, this work is a preliminary study focused on an in vitro model that demonstrates the ability of miR-369-3p to modulate PDE4B expression and its downstream inflammatory cascade. Further studies are required to confirm the anti-inflammatory effects of miR-369-3p in IBD mouse models.

## 4. Materials and Methods

### 4.1. Cell Culture and miR-369-3p Mimic Transfection

The Raw264.7 cell lines were obtained from American Type Culture Collection (ATCC, Manassas, VA, USA). The cells were used between passages 7 and 15 and cultured in Dulbecco’s modified Eagle medium (DMEM, Thermo Fisher Scientific, Waltham, MA, USA) supplemented with 10% heat-inactivated fetal bovine serum (FBS, Thermo Fisher Scientific, Waltham, MA, USA), 1% 10,000 µg/mL streptomycin and 10,000 U/mL penicillin (Thermo Fisher Scientific, Waltham, MA, USA), 1% 1M HEPES (Sigma-Aldrich, St. Louis, MO, USA), and 1% 100 mM sodium pyruvate (Sigma-Aldrich, St. Louis, MO, USA). Cells were incubated at 37 °C in a humidified atmosphere with 5% CO_2_.

When Raw264.7 cells reached confluence, they were harvested and seeded into 12-well plates at a density of 1 × 10^6^ cells/well. After 24 h, cells were transfected with miR-369-3p mimic (Life Technologies, Hilden, Germany) at a concentration of 30 and 50 nM using the TransIT-TKO Transfection Reagent (Mirus Bio LLC, Madison, WI, USA) following the manufacturer’s instructions. Cells were then stimulated with lipopolysaccharide (LPS, Sigma-Aldrich, St. Louis, MO, USA) at the final concentration of 1 μg/mL for 6 h and then lysed for RNA isolation and protein extraction. Each experimental condition was associated with a mock control consisting of cells treated with the same transfection reagent but without miRNA mimic.

### 4.2. RNA Extraction and Real-Time PCR

Total RNA was isolated using TRIzol reagent (Invitrogen, Carlsbad, CA, USA) according to the manufacturer’s protocol. The total RNA concentration was determined using the NanoDrop spectrophotometer (Thermo Fisher Scientific, Waltham, MA, USA). cDNAs were obtained from 1 μg of total RNAs using the iScript Reverse Transcription Supermix (BioRad Laboratories, Hercules, CA, USA) based on the manufacturer’s instructions. qPCR amplification reactions were conducted on a CFX96 System (Biorad Laboratories, Hercules, CA, USA) using SsoAdvanced Universal SYBR Green Supermix (BioRad Laboratories, Hercules, CA, USA) and the primers QuantiTect Primer Assay for Pde4B and Gapdh (Qiagen, Hilden, Germany). Gapdh gene amplification represented the housekeeping control used to normalize the Pde4b gene expression. Comparative real-time PCR was performed in triplicate.

### 4.3. Protein Isolation and Immunoblotting Analysis

Cells were lysed with T-PER Protein Extraction Reagent (Thermo Fisher Scientific, Waltham, MA, USA) enhanced by a protease inhibitor cocktail (Sigma-Aldrich, St. Louis, MO, USA). The protein concentration was determined using Bradford’s protein assay (Biorad Laboratories, Hercules, CA, USA). Equal protein concentrations were subjected to SDS-PAGE on 4–20% Mini-PROTEAN TGX Stain-Free Protein Gels (Biorad Laboratories, Hercules, CA, USA) and were transferred onto PVDF membranes (Biorad Laboratories, Hercules, CA, USA). For protein detection, membranes were incubated in iBind Automated Western Systems (Thermo Fisher Scientific, Waltham, MA, USA) following the manufacturer’s instructions. The primary antibodies used were rabbit mAb anti-PDE4B (ab170939, Abcam, Cambridge, UK, dilution 1:1000), rabbit mAb anti-pCREB (#9198, Cell Signaling, Technology, Danvers, MA, USA, dilution 1:1000), rabbit mAb anti-CREB (#9197, Cell Signaling, Technology, Danvers, MA, USA, dilution 1:1000), rabbit polyAb anti-PKA C-α (#4782, Cell Signaling, Technology, Danvers, MA, USA, dilution 1:1000), mouse mAb anti-GAPDH (sc-365062, Santa Cruz Biotechnology, Inc., Heidelberg, Germany, dilution 1:1000), and secondary antibodies goat anti-mouse IgG-(H+L)-HRP conjugate (170-6516, Biorad Laboratories, Hercules, CA, USA, dilution 1:500) and goat anti-rabbit IgG-(H+L)-HRP conjugate (31466, Invitrogen, Carlsbad, CA, USA, dilution 1:2500). Membranes were imaged using the ChemiDoc Imaging System (Biorad Laboratories, Hercules, CA, USA), analyzed with Image Lab Software version 5.2.1 (Biorad Laboratories, Hercules, CA, USA), and quantified by ImageJ Software 1.54d. GAPDH expression was used as a housekeeping control to normalize the protein expression.

### 4.4. Measurement of Cytokine Production

For cytokines analysis, the supernatants were collected after transfection and LPS stimulation. The analysis of IL-1β, TNF-α, IL-10, IL-1α, and IL-1Rα was conducted using ELISA (R&D Systems, Minneapolis, MN, USA) kits in accordance with the manufacturer’s instructions. The supernatants were transferred into 96-microwell plates (Costar Corning, Corning, NY, USA) coated with specific capture antibody. Then, the captured cytokine was detected with a detection antibody. Subsequently, the binding was measured after addition of streptavidin–horseradish peroxidase conjugate, and the reaction was then developed with the TMB. The plates were read using the SPECTROstar Omega Spectrometer (BMG Labtech, Ortenberg, Germany) at an absorbance of 450 nm.

### 4.5. Immunofluorescence

Raw264.7 cells were seeded in Lab-Tek Chamber Slides (Thermo Fisher Scientific, Waltham, MA, USA) at a density of 2 × 10^5^ cell/well. Following transfection and LPS stimulation, cells were washed twice with PBS and fixed with 4% paraformaldehyde (PFA, Sigma-Aldrich, St. Louis, MO, USA) for 15 min. Subsequently, they were washed three times with PBS, and cells were permeabilized and blocked in PBS + BSA 3% and Triton-X 0.1% for 1 h at room temperature. Next, cells were stained with the primary antibodies rabbit monoclonal cAMP (ab134901, Abcam, Cambridge, UK, dilution 1:100) and mouse monoclonal PDE4B (MA-25677, Thermo Fisher Scientific, Waltham, MA, USA, dilution 1:100) in PBS + BSA 3% overnight at 4 °C. Then, cells were incubated with the secondary antibodies chicken anti-mouse IgG (H+L) Alexa Fluor 488 (A21200, Invitrogen, Carlsbad, CA, USA, dilution 1:400) and chicken anti-rabbit IgG (H+L) Alexa Fluor 594 (A21442, Invitrogen, Carlsbad, CA, USA, dilution 1:400) in PBS + BSA 3% for 1h at room temperature. After washing with PBS, ProLong Gold Antifade Mountant with DAPI (Thermo Fisher Scientific) was applied and mounted with a glass cover slip. Images were assessed using a fluorescence microscope (Eclipse Ti2, Nikon Inc., Melville, NY, USA) using filters for DAPI, RFP/TRITC, and GFP/FITC.

### 4.6. Immunohistochemistry in UC Samples

Paraffin-embedded tissue blocks consisting of intestinal surgical resections from patients with UC and without UC were obtained from 30 patients subdivided into two groups: the UC group (ulcerative colitis, *n* = 20 patients) and the HC group (healthy control, *n* = 10 controls).

To confirm the adequacy and morphologic and/or pathological characteristics of the samples, sections stained with hematoxylin and eosin were reviewed by a pathologist. For IHC analysis, 4 µm sections were cut and mounted on Apex Bond IHC slides (Leica Biosystems, Buffalo Grove, IL, USA). IHC staining procedures were performed on the BOND III automated immunostainer (Leica Biosystems, Buffalo Grove, IL, USA). Tissue sections were incubated with anti-PDE4B primary antibody (MA-25677, Thermo Fisher Scientific, Waltham, MA, USA, dilution 1:1000). The Bond Polymer Refine Detection Kit (Leica Biosystems, Buffalo Grove, IL, USA) was used as a chromogen reagent according to the manufacturer’s instructions. Samples were negative when the number of stained cells was less than 5%.

The expression of PDE4B was determined through a score calculated as follows: 0, absent; 1, weak expression; 2, moderate expression; 3, intense expression.

### 4.7. Bioinformatics and Statistical Analysis

Putative gene targets of miR-369-3p were predicted by the miRDB (https://www.mirdb.org/ accessed on 20 September 2023) [42], miRmap (https://mirmap.ezlab.org/ accessed on 21 September 2023) [43], and TargetScan (https://www.targetscan.org/mmu_72/ accessed on 22 September 2023) [44] algorithms.

Data were evaluated using GraphPad Prism software version 10.0.2 and expressed as the mean ± SEM. According to the normal distribution of data, statistical significance was evaluated with a two-tailed Student’s *t* test or Mann–Whitney. Data were derived from at least four independent experiments. Differences among experimental conditions were considered statistically significant at *p* < 0.05.

## 5. Conclusions

In conclusion, achieving augmented intracellular cAMP levels by inhibiting PDE4B has been revealed as a good therapeutic strategy to mitigate inflammation in several inflammatory diseases. Our results support the ability of miR-369-3p to act in this direction by directly targeting PDE4B as well as mediating downstream cAMP signaling. Because high levels of PDE4B are present in UC patients, we provide the basis for a greater understanding of inflammatory signaling in UC, suggesting that miR-369-3p, by ameliorating inflammatory conditions, could be used as a new therapeutic approach to inflammatory bowel disease.

## 6. Patents

An Italian patent entitled “Pharmaceutical composition based on miR-369-3p as active ingredient for the treatment of chronic inflammatory disorders” (patent n° 102018000007954) was issued on 3 August 2020 to the Ente Ospedaliero Specializzato in Gastroenterologia “Saverio de Bellis”.

## Data Availability

Data are within the article.
